# RETRACTION: Up‐regulation of KISS1 as a Novel Target of Let‐7i in Melanoma Serves as a Potential Suppressor of Migration and Proliferation In Vitro

**DOI:** 10.1111/jcmm.71364

**Published:** 2026-09-08

**RETRACTION:** H. A. Alkafaji, A. Raji, H. S. Rahman, A. O. Zekiy, A. Adili, M. Jalili, T. Hojjatipour, A. Cid‐Arregui, N. Shomali, S. Tarzi, R. Tamjidifar, R. Heshmati, F. Marofi, M. Akbari, A. Hasanzadeh, M. Deljavanghodrati, M. Jarahian, S. S. Shotorbani, “Up‐regulation of KISS1 as a Novel Target of Let‐7i in Melanoma Serves as a Potential Suppressor of Migration and Proliferation In Vitro”, *Journal of Cellular and Molecular Medicine* 25, no. 14 (2021): 6864‐6873, https://doi.org/10.1111/jcmm.16695.

The above article, published online on 6th June 2021 on Wiley Online Library (https://onlinelibrary.wiley.com), has been retracted by agreement between the authors, Editor‐in‐Chief Stefan N. Constantinescu, the Foundation for Cellular and Molecular Medicine, and John Wiley and Sons Ltd. The article has been retracted because essential information about experimental methods is missing and therefore the Editors do not consider the results to be reproducible.

